# A Case of Addison’s Disease

**Published:** 1878-01-20

**Authors:** 


### *A CASE OF ADDISON'S DISEASE.*

In an article on this disease published in *The American Journal of the Medical Sciences,* for January, 1877, Dr. William Pepper states that only ten cases of Addison's disease are quoted from American sources in Greenhow's tables. I therefore desire to put on record the only case I have met with, although the notes are not so full as could be wished. The family attendant, an eclectic physician, now dead, kindly furnished me with an account of the symptoms noted before death.

On the 8th of August, 1869, I was asked to make an autopsy of Ida S., aged seventeen years, who died the day before. Her mother says that the patient's menses have been regular since she was thirteen years old. Her complexion began to grow dark a year before; others of the family think this date is placed too far back, but Mrs. S. insists that twelve months have passed since the change was noted. The girl's health began at that time to fail, but so insidiously that she was not considered ill till five months previously. Just before that time I saw her while attending her grandfather for a railroad accident which proved fatal, but only noticed that she was darker than the rest of the family, and was not asked for advice. Early in March, she showed a dark circle around the neck, which was supposed to be caused by some article of dress, and the discoloration began to be observed by others beside her mother. Along with this she began to be puffy under the eyelids in the morning, was easily fatigued and rendered breathless, had frequent nausea and vomiting, headache, backache, and "sideache." Her appetite became capricious, with craving for salt and acids; the bowels were generally constipated, with occasional diarrhœa, languor and sleepiness, and latterly hiccough. She had been up and about the house during this illness, and only a week before her death she walked out. It was not till the 3d of August that this practitioner was called to see her; he was surprised by the discoloration of the skin, which was of the hue of a mulatto over the whole body, deepening almost to black in the folds of joints. She had pain and great tenderness in the lumbar region, increased by pressure; the menses were present. The next day vomiting set in, and continued till 3 o'clock in the morning of the 6th, with relief to the pain in the back; an enema produced a copious natural discharge. The thirst was insatiable. No tenderness of the epigastrium was observed, but great distress and distention before a fit of vomiting, which relieved it. There was no headache, but slight delirium; no convulsions; the breathing occasionally stertorous. Twelve ounces of urine were passed, the constituents of which are not recorded. The region of the liver was flattened, not tender. Exhalations from the skin were fœtid, a very bad odor being constantly in the room; there were no hemorrhages.

August 6th. The vomiting, stopping at three a. m., recommenced at five, with constant nausea.

August 7th, eight a. m. Nausea ceased during the night, and the patient said she felt better; tongue partly cleaned; skin mottled and streaked. Slight trismus was noticed while her mouth was washed with cold water; the conjunctivæ were congested; there was slight delirium. Died at 10 o'clock a. m.

Autopsy, August 8th, at 3:30 p. m., by Dr. Stedman. Color, that of a light mulatto, having cleared very much since death. Body slight, but not emaciated. Lips livid, as if she had been eating mulberries. Mammary development very large for a young American girl; areolæ almost black, and shining cracks in the skin of the breasts; nipples very small and undeveloped; otherwise the appearance of the breasts would have suggested pregnancy. Head not opened. Heart small and flabby, not fatty. Lungs crepitant throughout, but firmly glued to costal pleuræ and diaphragm by old adhesions. Stomach large, and its walls thin. The coats of the intestines thin; abdominal glands somewhat enlarged. Liver enlarged, flabby; the right lobe looking fatty, adherent by its lower surface, requiring dissection to free it; in doing this an abscess was cut into on either side; these were seated in the supra-renal capsules, which adhered to the liver, were of firm texture, and the size of a man's thumb, holding a drachm of pus-like fluid, and the remaining substance looked like broken down caseous matter. The uterus slightly anteflexed and virginal. Other organs were examined and found normal. —*C. Ellery Stedman, in The Boston Medical and Surgical Journal.*

